# Insights into artificial intelligence-based digital pathology in hepatobiliary and pancreatic cancer

**DOI:** 10.3389/fonc.2026.1871770

**Published:** 2026-09-08

**Authors:** He-Yu Huang, Alfred Wei Chieh Kow, Zhong-Qi Fan, Guo-Yue Lv

**Affiliations:** 1 Department of Hepatobiliary and Pancreatic Surgery, General Surgery Center, First Hospital of Jilin University, Changchun, Jilin, China; 2 China-Singapore Belt and Road Joint Laboratory on Liver Disease Research, First Hospital of Jilin University, Changchun, Jilin, China; 3 Division of Hepatobiliary & Pancreatic Surgery, Department of Surgery, National University Hospital Singapore, Singapore, Singapore

**Keywords:** artificial intelligence, cholangiocarcinoma, hepatocellular carcinoma, pancreatic cancer, precision medicine

## Abstract

Artificial intelligence (AI)-driven digital pathology has emerged as a promising approach for improving diagnosis, prognostic assessment, and treatment decision-making in hepatobiliary and pancreatic cancers (HPCs). By leveraging whole-slide imaging and deep learning, AI enables high-throughput and reproducible analysis of complex histopathological data, facilitating precision oncology. This review summarizes recent advances in AI-assisted digital pathology across hepatocellular carcinoma, biliary tract cancer, and pancreatic cancer, with a focus on diagnostic applications, molecular prediction, prognostic modeling, and treatment response assessment. Despite rapid technical progress, the clinical translation of AI remains limited. Current challenges extend beyond model performance and include data heterogeneity, limited interpretability, lack of prospective validation, and insufficient integration into clinical workflows. Notably, the central limitation lies in the misalignment between algorithm development and clinically relevant problem definition. Future research should prioritize multicenter validation, multimodal data integration, and the development of biologically informed and clinically interpretable models. Importantly, AI is expected to evolve from a diagnostic support tool to a dynamic, workflow-integrated decision-support system. Overall, the impact of AI-driven digital pathology will depend not only on technological advancement, but on its ability to deliver measurable clinical benefit and to be effectively embedded into real-world clinical practice.

## 1 Introduction

Histopathology, as the gold standard for disease diagnosis, traditionally relies on optical microscopy, combined with cellular and molecular information when necessary. However, increasing demands for precision and efficiency in modern oncology have exposed important limitations of conventional pathology workflows. In recent years, the rapid development of high-throughput scanning technologies and equipment has led to the widespread adoption of digital pathology in laboratories, with the potential to revolutionize clinical diagnostic workflows. However, challenges remain in terms of implementation costs, standardization, data privacy, network efficiency, and legal regulations, which still require further optimization (1). Overall, despite clear advantages, large-scale clinical implementation remains constrained by practical and regulatory barriers.

Since 2012, AI has advanced rapidly in the field of medical image analysis (2). Evidence suggests that AI can be effectively applied across histopathology, radiology, and clinical data, with early clinical applications already emerging in selected cancer types. The integration of AI with digital pathology has the potential to overcome existing limitations by extracting richer information from pathological images. Some studies have even indicated that AI’s diagnostic accuracy is comparable to, or better than, that of human experts (1, 3). However, the widespread clinical application of AI still faces challenges such as poor interpretability and insufficient robustness, which urgently need to be addressed (3, 4). Importantly, the central challenge is no longer model performance alone, but the translation of these advances into clinically reliable applications. Notably, the regulatory dimension of this translation challenge—including the pathway to clinical approval and the current absence of HPC-specific approved tools—remains underexplored.

**Figure 1:**
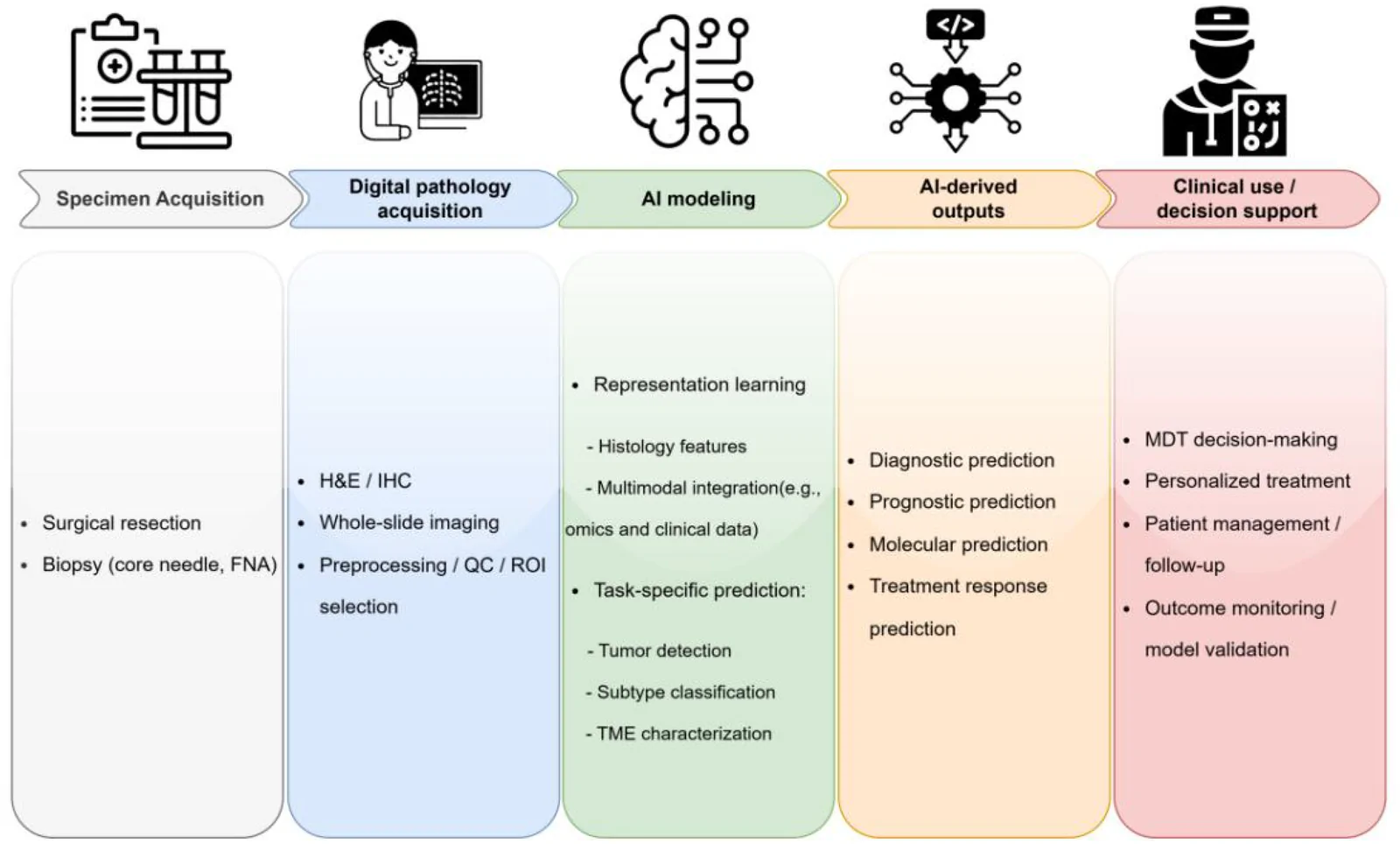
Conceptual framework of AI-driven digital pathology in hepatobiliary and pancreatic cancers. This schematic illustrates the end-to-end conceptual workflow of AI-driven digital pathology, including specimen acquisition (surgical resection or biopsy), digital pathology processing (H&E/IHC staining, whole-slide imaging, and preprocessing), AI-based analysis (representation learning with multimodal integration and task-specific prediction), and clinical integration. The framework highlights how AI-derived outputs support diagnostic, prognostic, molecular, and treatment response prediction, ultimately enabling clinical decision-making, personalized treatment, and outcome monitoring in precision oncology. HCC, hepatocellular carcinoma; BTC, biliary tract cancer; PC, pancreatic cancer.

Hepatobiliary and pancreatic cancers (HPCs) are common malignancies of the digestive system, often characterized by insidious onset and late-stage diagnosis, leading to high mortality rates (3). These features make HPCs particularly suitable for AI-assisted approaches aimed at improving diagnostic accuracy and prognostic stratification. Therefore, timely and accurate diagnosis, along with precise prognostic analysis, is crucial for optimizing treatment and improving patient outcomes. In this review, we summarize recent advances, translational potential, and emerging applications of AI-driven digital pathology in HPCs, with the aim of providing insights into future research directions and key challenges. Importantly, AI-driven digital pathology is evolving beyond pattern recognition toward clinically actionable decision support, bridging histopathological features with prognosis, molecular characteristics, and therapeutic response. As illustrated in **Figure 1**, we conceptualize AI-driven digital pathology as a structured, end-to-end workflow encompassing specimen acquisition, digital pathology processing, AI modeling, and clinical integration, thereby linking computational outputs to real-world clinical decision-making (1) (**Figure 1**). Taken together, this framework highlights a shift from descriptive image analysis toward clinically integrated, decision-oriented AI systems.

This review is based on a literature search of PubMed and Web of Science conducted through January 2026, using terms related to artificial intelligence, digital pathology, and hepatobiliary and pancreatic cancers. Studies were selected based on relevance to the topics covered.

## 2 Applications of AI-driven digital pathology in HCC

### 2.1 An overview of diagnostic and decision-making workflow for liver lesions

As outlined in the general framework (**Figure 1**), the comprehensive process of liver cancer management involves but is not limited to disease prevention, early diagnosis, optimization of therapeutic strategies, and prognosis assessment. AI plays a pivotal role at each stage, supporting precision medicine.

Pathological specimens for HCC primarily originate from fine-needle aspiration (FNA) and surgical excisions. Routine evaluation involves hematoxylin and eosin (H&E) staining and immunohistochemical staining (IHC), with molecular testing being conducted when necessary. However, widespread implementation of molecular testing is limited due to high cost and limited accessibility. The core responsibilities of pathologists include diagnosis and differential diagnosis, such as distinguishing between benign and malignant lesions, identifying tumor types, and assessing TNM staging. Additionally, pathologists can determine tissue and cell subtypes based on the WHO classification system, although this is more commonly applied in research settings than in clinical practice.

Therapeutic strategies, including surgery, targeted therapy, immunotherapy, chemotherapy, or radiotherapy, are formulated based on pathological findings and individual patient characteristics. Overall, the diagnostic and treatment pathway for liver lesions represents a multidisciplinary and stepwise process, in which AI-driven digital pathology has the potential to enhance diagnostic accuracy, improve risk stratification, and support clinical decision-making across multiple stages. Among hepatobiliary and pancreatic cancers, AI-driven digital pathology is most extensively studied in HCC, where applications have evolved from diagnostic assistance to multimodal prognostic and therapeutic prediction.

### 2.2 AI-driven digital pathology in the diagnosis of HCC

Although HCC diagnosis can be based on patient records (*e.g.* viral hepatitis, excessive alcohol consumption, or cirrhosis), imaging features, and laboratory findings, histopathology remains the gold standard. Imaging analysis, however, is inherently qualitative and prone to errors (4). With the growing use of immunotherapy and targeted therapies, current guidelines recommend biopsies to analyze tumor immune characteristics, guiding treatment decisions (5, 6). Against this background, deep learning applications in liver tumor pathology have rapidly expanded.

Digital pathology images, characterized by their complex structure and billions of pixels, provide an ideal platform for deep learning, which excels in analyzing such large datasets. Deep learning has significantly improved the efficiency and accuracy of liver tumor diagnosis (7). In representative cohorts, deep learning models have demonstrated strong performance in classifying hepatic nodular lesions, achieving AUCs around 0.90–0.94 while reducing pathologist workload (8).

For patients with malignant liver lesions, distinguishing HCC from CCA is essential for choosing therapeutic options. Deep learning models trained on multi-cohort datasets have achieved accurate differentiation between HCC and CCA (9). However, diagnosing rare tumors such as cHCC-CCA remains challenging due to complex histological features. Recent WSI-based models have demonstrated the ability to reclassify cHCC-CCA into HCC or iCCA with strong correlation to molecular profiles (10).

In the diagnosis of iCCA, one of the major challenges is distinguishing primary liver lesions from metastatic ones, particularly those originating from colorectal cancer. Accurate differentiation is critical for making clinical decision (11). Recent studies report that deep learning models can differentiate iCCA from colorectal liver metastases with high accuracy (up to ~98% in external validation), outperforming some pathologists (12). Another study developed a deep learning model capable of distinguishing HCC, CCA, and mCRC simultaneously. While initial performance on external datasets was suboptimal, the integration of additional data and retraining the model significantly improved the model’s accuracy, offering directions for future research (13). Furthermore, deep learning has been employed to predict the primary site of metastatic tumors or cancers of unknown primary. A related study reported an AUC of 83% in external validation cohorts, highlighting the potential of this approach to narrow down differential diagnoses for liver metastases, providing clinicians with more precise assistance (14) (**Table 1**).

**Table 1:** Representative studies on AI-driven digital pathology for the diagnosis and differential diagnosis of hepatocellular carcinoma.

Year	Author	Modality	Model	Sample size	Application
2020 (9)	Kiani et al.	Histopathology	CNN	Combined total 70; external validation 80	Classify HCC vs CCA
2021 (14)	Lu M Y et al.	Histopathology	CNN	Combined total 29807; Internal test 6499; External validation 682	Distinguish tumors as primary or metastatic, and predict the primary site of metastatic tumors
2022 (8)	Na Cheng et al.	Histopathology	CNN	Discovery cohort: 330; validation:66	Differentiate every kind of HNLs
2023 (10)	Julien Calderaro1 et al.	Histopathology	CNN	Discovery cohort: 424(HCC)167(iCCA); Validation cohort: 40(cHCC-CCA)	Differentiate cHCC-CCA into HCC or iCCA
2023 (12)	Albrecht et al.	Histopathology	CNN	Combined total 571; external validation 159	Differentiate iCCA from colorectal liver metastasis
2023 (13)	Jang H-J et al.	Histopathology	CNN	Discovery cohort: 366(HCC)195(CC)179(mCRC); Validation cohort: 31(HCC)29(CCA)	Differentiate HCC from CCA and mCRC

CNN, convolutional neural network; HCC, hepatocellular carcinoma; cHCC-CCA, combined hepatocellular-cholangiocarcinoma; iCCA, intrahepatic CCA; HNLs, hepatic nodular lesions; mCRC, metastatic colorectal cancer.

Beyond histopathology, emerging blood-based molecular approaches, including microbial cell-free DNA profiling, may provide complementary information for differentiating primary HCC from colorectal liver metastases (15).

In the field of liver diseases, AI plays a critical role in both digital pathology and imaging, with each having distinct focuses. Digital pathology, with its wealth of microstructural information such as cell distribution and nuclear morphology, surpasses imaging in terms of data richness and predictive capability. It also provides deeper insights into the molecular alterations associated with tumors (16), whereas radiomics relies on pathology as a reference.

Overall, AI-driven digital pathology offers substantial scientific and translational value by improving diagnostic precision and supporting precision medicine (17).

### 2.3 AI-driven digital pathology for predicting gene mutations in HCC

Gene mutations are critical biomarkers in cancer immunotherapy, but their clinical application is limited by technical complexity, high costs, and tedious procedures (16). AI-based digital pathology has demonstrated the potential to overcome these limitations in cancer gene prediction (17). By analyzing digital pathology slides, AI can infer gene mutations, molecular subtypes, gene expression profiles, and pathological biomarkers (4). Morphological features in HCC have been shown to correlate with clinical outcomes and molecular alterations (18–23). Although initial genomic analyses, such as whole-genome sequencing and RNA sequencing, have shown promise in identifying therapeutic targets in advanced HCC, these methods are not yet widely implemented in clinical practice (24).

In addition to somatic mutations, HCC is characterised by extensive epigenetic alterations, including aberrant DNA methylation, histone modifications, chromatin remodelling and non-coding RNA dysregulation, which may also generate morphologically detectable phenotypes (25).

AI models have exhibited predictive capabilities across various tumor types, particularly in identifying gene alterations in *FGFR*, *IDH*, *PIK3CA*, *BRAF*, *TP53*, and DNA repair pathways (26). Representative studies report that deep learning models can predict tumor mutational burden directly from histopathology with very high accuracy (AUC up to 0.99), outperforming conventional sequencing approaches (16). Subsequently, Fu et al. developed a transfer learning model that predicted *TP53* mutations in HCC with an AUC of 0.84 (95% CI: 0.80–0.88) (27). Additionally, Kather J N et al. built a pan-cancer model that identified *CTNNB1* as a relevant gene in liver cancer (28).

Additional studies using CNN-based approaches have demonstrated the ability to predict multiple gene alterations, including TP53, CTNNB1, ALB, TTN, and PCLO, with AUCs ranging from 0.71 to 0.89 (29, 30). Generative models such as HistoXGAN further suggest the feasibility of “virtual biopsies” that preserve tumor grade, histological subtypes, and gene expression patterns (31).

Taken together, AI-driven digital pathology provides a rapid, scalable, and cost-effective approach for molecular prediction. It is particularly suitable for pre-screening applications, reducing reliance on complex genomic assays while improving efficiency. Overall, these approaches highlight substantial translational potential for integrating image-based molecular prediction into clinical and research workflows (26).

### 2.4 AI-driven digital pathology for predicting tumor clonality and spatial heterogeneity in HCC

In recent years, advancements in spatial transcriptomics (32–36) and genomic technologies (37, 38) have provided powerful tools for studying tumor spatial heterogeneity. AI models leveraging whole-slide images and single-cell data have demonstrated strong capability in characterizing tumor clonality and spatial organization (39). These models, trained on genomics, transcriptomics, and prognostic data, enable spatially informed analysis and generate biologically meaningful insights (40).

For instance, Wang H et al. developed a multi-task deep learning neural network that enabled automated single-cell segmentation and classification on digital slides (41). This approach extracted spatial features related to the tumor microenvironment, which, through unsupervised clustering, identified three subtypes associated with specific genomic alterations and molecular pathways. In parallel, multiplexed imaging techniques such as CODEX have shown that spatial proximity between tumor cells and CD8+ T cells is associated with favorable prognosis in HCC (42).

Taken together, integration of AI with emerging spatial technologies is advancing the understanding of tumor heterogeneity. These approaches provide insights into tumor biology and offer potential for future translational applications.

### 2.5 Applications of AI-driven digital pathology in prognostic prediction for HCC

Basic applications of deep learning aim to automate workflows in pathology, including disease diagnosis, grading, and subtype classification, thereby reducing the workload of pathologists. Advanced applications focus on providing prognostic information, mutation analysis, and treatment response predictions to optimize therapeutic strategies (43). While many deep learning studies in histopathology primarily concentrate on cancer diagnosis (44), recent work has increasingly emphasized prognostic prediction in HCC (45).

Deep learning models have demonstrated improved prognostic performance compared with conventional clinical variables, with concordance indices around 0.70 in external validation cohorts (46). This underscores the importance of collaboration between pathologists and AI models in enhancing model performance. Shi et al. developed a weakly supervised deep learning framework using WSIs to identify an independent prognostic factor that outperformed existing clinical staging systems (47). The framework demonstrated strong generalizability across patient cohorts in China and the United States. Additionally, Liang et al. utilized a multi-class tissue segmentation network (PaSegNet) to generate spatial distribution probability heatmaps of WSIs. Using attribution methods, they proposed two independent clinical indices that outperformed traditional staging systems in prognostic prediction (48).

Despite these advancements, most prognostic models remain limited to single data sources, such as histology or genomics, without effectively integrating multimodal data (49). Multimodal approaches combining WSIs with molecular data have demonstrated improved prognostic accuracy and revealed associations between morphological features (e.g., tumor infiltration, stroma, necrosis) and molecular characteristics (50). As research advances, the focus is shifting from survival prediction to tumor recurrence. Yamashita R et al. developed a deep learning model that achieved an AUC of 90.8% in an external test set for tumor classification, enhancing the prediction of recurrence risk (51).

Recent studies have further explored microscopic features as prognostic markers. Quantification of reticulin framework alterations has been associated with metastasis and survival outcomes in HCC (52). Integration of histopathological features with clinical variables through multimodal frameworks has further improved predictive performance (53).

### 2.6 AI-driven digital pathology for predicting treatment response in HCC

The ability to predict treatment response holds greater clinical significance than traditional prognostic assessment, as it enables more precise therapeutic strategies (6). Current approaches often rely on indirect biomarkers. For example, microsatellite instability is used to assess response to immune checkpoint inhibitors across multiple cancers, with emerging relevance in HCC (54–61).

Zeng et al. proposed an AI-based approach to predict the activation levels of immune and inflammatory gene signatures directly from HCC histological samples (62). These signatures are associated with response to immunotherapy. Furthermore, they developed the ABRS-P model, which predicts response to first-line atezolizumab plus bevacizumab treatment directly from H&E-stained slides (63), providing a cost-effective alternative to molecular assays.

For treatment monitoring, deep learning models based on imaging data have also been explored. A model using CT images was developed to predict response to transarterial chemoembolization and assist in patient selection (64). However, the stability and generalizability of imaging-based biomarkers require further validation.

Direct prediction of treatment response from histopathology may offer greater clinical value than indirect biomarkers. However, AI-driven digital pathology for treatment monitoring remains underdeveloped, and most studies are based on retrospective cohorts with limited real-world validation.

## 3 Applications of AI-driven digital pathology in biliary tract cancer

### 3.1 AI-driven digital pathology in the diagnosis of BTC

AI integrated with digital pathology also plays a crucial role in the diagnosis of BTC, which includes intrahepatic cholangiocarcinoma (iCCA) and perihilar cholangiocarcinoma(pCCA) and gallbladder tumor (GBC) (65). In clinical practice, BTC diagnosis often relies on small biopsy or cytology specimens obtained through endoscopic or cholangioscopic procedures, posing challenges for AI model development and validation. As BTC originate from bile duct epithelial cells (66), accurate and timely diagnosis is essential for improving patient outcomes.

AI combined with digital pathology has demonstrated the ability to differentiate malignant liver lesions, including HCC and CCA (9), refine classification of mixed tumors such as cHCC-CCA (10), and identify the origin of hepatic lesions (12).

Regarding BTC, Sun L et al. developed a benchmark dataset of microscopic hyperspectral pathology images and showed that CNN architectures can effectively leverage spatial–spectral information for accurate detection and classification (67). Subsequently, Chakrabarti S et al. proposed a lightweight neural network (CholangioNet) for rapid identification of cholangiocarcinoma using histopathological RGB images (68), achieving improved performance compared with conventional architectures. Beyond H&E-based diagnosis, AI has been extended to IHC analysis for risk stratification. For example, Sjöblom et al. used keratin 7 staining to predict cholangiocarcinoma risk in patients with primary sclerosing cholangitis (69).

Overall, compared with HCC, AI applications in BTC remain at an earlier stage, largely due to limited data availability and tumor heterogeneity, with most studies focusing on proof-of-concept rather than clinically actionable applications.

Currently, research on the integration of artificial intelligence with digital pathology in BTC remains insufficient (70). Existing studies mainly focus on BTC as a whole, whereas research specifically on GBC is still scarce. Collaborative efforts among researchers from high-incidence regions of GBC are essential to address this gap.

### 3.2 Applications of AI-driven digital pathology in predicting gene mutations in BTC

Research on molecular biomarkers for CCA has progressed substantially, offering new hope for patients with progression after first-line therapy. Studies indicate that approximately 76% of CCA patients harbor actionable genomic alterations, nearly half of whom have benefited from targeted therapies (24). Common alterations include FGFR2 rearrangements, IDH1/2 mutations, and BRAF V600E in iCCA, as well as HER2 amplification and EGFR mutations in GBC and pCCA. In addition, rarer events such as NTRK fusions, VEGFR-related pathways, and immune checkpoint markers (PD-1, PD-L1, CTLA-4, LAG-3) remain important therapeutic targets (71).

Furthermore, studies indicate that non-coding RNAs, including miRNAs, lncRNAs, and circRNAs, have potential roles in early diagnosis, prognostic assessment, and personalized treatment of BTC (72). Integration of AI with multi-omics data may improve ncRNA-based classification and biomarker discovery in CCA. However, evidence in this area remains limited.

Overall, although genomics-guided approaches show promise in BTC, their clinical application remains limited. Large-scale, multicenter studies are required to establish broader clinical applicability.

### 3.3 Applications of AI-driven digital pathology in predicting prognosis of BTC

AI combined with digital pathology has demonstrated significant potential in predicting prognosis for BTC. However, due to the low incidence of CCA, constructing large-scale patient cohorts for deep learning studies remains challenging, and related research is still relatively limited.

Foundation models pretrained on large multi-cancer datasets offer a transfer learning strategy to overcome this cohort-size limitation, enabling effective fine-tuning on small BTC-specific datasets (see Section 5.1) (73, 74).

Xie et al. developed a model to predict survival in iCCA patients, achieving an AUC of 0.6818 in an independent test set (*P*-value = 0.03) (75). Their findings indicate that spatial organization of tumor and immune components is associated with survival differences. Additionally, Ding et al. focused on tertiary lymphoid structures (TLS), creating a computer-assisted TLS scoring system that revealed contrasting prognostic roles of intratumoral and peritumoral TLS (76).

Unsupervised and feature-based approaches have further identified histological patterns associated with disease progression. For example, an unsupervised deep learning model identified fibrosis-related collagen deposition as a marker of disease severity (77). Multimodal models integrating histological and molecular data have also identified key pathways (e.g., glycolysis, hypoxia, mTORC1 signaling, immune infiltration) associated with prognosis (78).

Hoyer D P et al. attempted to combine clinical data, surgical details, pathological findings, and AI-based WSI analysis to develop a prognostic model for patients with pCCA (79), although performance remains modest. In imaging studies, Song et al. developed a multicenter model to predict early recurrence after iCCA resection, achieving AUCs of 0.871–0.882 and outperforming the AJCC TNM staging system (80). A summary of representative studies on diagnosis and prognosis in BTC is presented in **Table 2**.

**Table 2:** Research selection on applications of AI-driven digital pathology in diagnosis and prognosis of BTC.

Year	Author	Modality	Model	Sample size	Application	Key findings
2021 (67)	Sun et al.	Histopathology	CNN	Total Sample Size: 880 microscopic hyperspectral image; Training Set: 580 images; Test Set: 150 images	Diagnosis	Enables spatial–spectral classification of CCA
2023 (68)	Chakrabarti et al.	Histopathology	CNN	Total Sample Size: 15, 000 images; Training Set: 12, 000 images; Test Set: 3, 000 images	Diagnosis	Lightweight model improves diagnostic efficiency
2019 (77)	Hassan Muhammad et al.	Histopathology	CNN	Total Sample Size: 246 WSIs from iCCA patients who underwent surgical resection	Prognosis	Unsupervised patterns capture fibrosis-related progression
2022 (75)	Jiawei Xie et al.	Histopathology	CNN	Total Sample Size: 127 patients; Training Set: 78 cases; Test Set: 49 cases	Prognosis	Spatial TME features associate with survival
2024 (78)	Guang-Yu Ding et al.	Histopathology	CNN	Total Sample Size: 941 iCCA patients; Training Set: 373 patients; Internal Validation Set: 213 patients; External Validation Set: 168 patients; External Validation Set: 187 patients with available multi-omics data	Prognosis	Multimodal integration reveals biology–prognosis links
2024 (79)	Dieter P. Hoyer et al.	Histopathology	CNN	Total Sample Size: 142 pCCA patients; Training Set: 115 cases; Test Set: 27 cases	Prognosis	Demonstrates feasibility of AI in pCCA prognosis

Key findings summarize the principal contribution of each study to diagnosis or prognostic modeling.

CNN, convolutional neural network; PSC, primary sclerosing cholangitis; WSIs, whole-slide images; iCCA, intrahepatic CCA; pCCA, perihilar cholangiocarcin.

### 3.4 Applications of AI-driven digital pathology in predicting treatment response for BTC

In incurable HCC, studies have demonstrated the ability to directly predict response to first-line therapy (atezolizumab plus bevacizumab) from H&E-stained pathological slides (63). However, similar approaches in CCA remain limited due to insufficient response data, particularly for durvalumab combined with chemotherapy in advanced-stage patients (81, 82).

Genomic analysis plays a critical role in treatment decision-making for advanced iCCA. Specific molecular alterations, such as *FGFR2* fusions and *IDH1/2* mutations, are essential targets for personalized therapies. Additionally, biomarkers related to resistance, including transport proteins and *PD-L1* expression, further guide individualized treatment strategies (83).

Integration of AI with multimodal data (pathology, imaging, genomics, and proteomics) may facilitate the development of novel biomarkers for precision therapy in CCA (71).

Overall, although these advances highlight the emerging role of AI-driven digital pathology in BTC, significant challenges remain before clinical implementation. Current studies are constrained by small and heterogeneous datasets, limiting generalizability and external validation. Compared with HCC, AI applications in BTC are still at an earlier stage and largely focused on proof-of-concept rather than clinically actionable applications.

## 4 Applications of AI-driven digital pathology in pancreatic cancer

### 4.1 Applications of AI-driven digital pathology in the diagnosis of PC

AI-driven digital pathology has shown increasing potential in the diagnosis of pancreatic cancer, particularly pancreatic ductal adenocarcinoma (PDAC), which represents the vast majority of pancreatic malignancies. In clinical practice, the diagnosis of pancreatic lesions often relies on small biopsy or cytology specimens obtained through endoscopic ultrasound-guided fine-needle aspiration (EUS-FNA) or fine-needle biopsy (EUS-FNB). These samples are frequently limited in size, fragmented, and subject to sampling bias, posing significant challenges for both pathological assessment and AI model development. Against this background, deep learning-based approaches have been explored to improve diagnostic accuracy and efficiency. PDAC is one of the most common gastrointestinal malignancies and remains highly lethal, with a 5-year survival rate of approximately 10–12% (84, 85). Current imaging technologies have limitations in the early detection, diagnosis, and staging of PDAC (86). AI-based approaches have demonstrated improved diagnostic accuracy in multiple settings (87). Beyond PDAC, AI has also been applied to pancreatic cystic lesions, which remain a major diagnostic challenge. Accordingly, this section discusses three major diagnostic applications of AI-driven digital pathology in PC: cystic lesion classification, biopsy-based diagnosis, and automated PDAC detection.

Pancreatic cystic tumors are heterogeneous, with varying malignant potential, making accurate differentiation essential. Song et al. developed a system to distinguish serous from mucinous cystadenomas based on morphological features, including cystic structure and cellular density (88). Similarly, neural network models have achieved high performance in differentiating benign and malignant cystic lesions, with AUCs up to 0.96, outperforming conventional biomarkers and cytology (89). These findings suggest that AI enables quantitative characterization of cyst architecture beyond conventional assessment, facilitating improved risk stratification.

Accurate characterization of precursor lesions, particularly intraductal papillary mucinous neoplasms and PanIN-associated histological changes, may improve risk stratification and facilitate early clinical intervention (90). In biopsy-based diagnosis, EUS-FNA and FNB are widely used for suspected PC (91, 92). AI models enhance diagnostic performance by analyzing cytological and histological features. Representative studies report very high diagnostic accuracy, including near-perfect classification of benign and malignant cells and AUCs approaching 0.98 in FNA-based models (93). Additional models have achieved >90% accuracy in tissue classification and up to 87% accuracy in tumor microenvironment analysis using multiplex approaches (94, 95). These findings highlight the potential of AI for analyzing small and fragmented biopsy samples. However, variability in sample quality and lack of standardized acquisition protocols remain major challenges. Different specimen types—including cytology (FNA), core biopsy (FNB), and surgical resections—present distinct analytical challenges for AI models.

The increasing detection of incidental pancreatic lesions presents an opportunity for AI-assisted risk stratification, particularly when imaging, cytopathology and emerging liquid-biopsy biomarkers are interpreted within an integrated diagnostic framework (96).

In automated PDAC diagnosis, deep learning models have demonstrated strong performance in classification and segmentation tasks, with some approaches performing comparably to expert pathologists (97, 98). AI has also been applied to other pancreatic neoplasms, such as pNETs, where automated Ki67-based grading reduces subjectivity and workload (99). Beyond classification, recent studies increasingly integrate histopathological, molecular, and clinical data to improve diagnostic precision and risk stratification. Such multimodal approaches are likely to further enhance clinical applicability.

Taken together, AI-driven digital pathology in pancreatic cancer is gradually moving beyond proof-of-concept pattern recognition toward potential clinical decision support. By improving diagnostic accuracy in limited and heterogeneous samples, AI may facilitate more precise pathological assessment and risk stratification. However, prospective validation, standardized workflows, and integration into clinical practice remain essential before widespread implementation.

### 4.2 Applications of AI-driven digital pathology in predicting prognosis and treatment response for PC

Similarly, Avram et al. demonstrated that multimodal models combining clinical, radiomic, and genomic features yielded the highest accuracy for predicting overall survival and recurrence in PDAC (100), with the use of explainable AI to improve interpretability.

AI-driven digital pathology enables automated analysis of histopathological images and supports prediction of survival and treatment response in PC. It also facilitates biomarker discovery and contributes to precision medicine.

Overall, although recent advances highlight the emerging role of AI in pancreatic cancer, several challenges limit clinical translation. Pancreatic cancer presents unique difficulties due to lesion heterogeneity and diverse sample types, including biopsy, cytology, and surgical specimens. Most studies remain retrospective and lack integration with longitudinal clinical data.

Pathology foundation models trained on diverse multi-cancer corpora present a promising strategy to overcome data scarcity in PDAC by enabling label-efficient transfer learning (see Section 5.1) (101, 102).

Furthermore, integration of AI into real-world clinical workflows—such as endoscopic sampling, early detection, and differentiation of cystic lesions—remains insufficiently explored. Given the aggressive nature of PDAC, future studies should prioritize clinically actionable applications, including early detection, treatment stratification, and response prediction.

Importantly, transitioning from isolated image-based models to multimodal, clinically integrated systems will be essential to improve patient outcomes.

## 5 Discussion

### 5.1 Why AI-driven pathology has not yet translated into routine clinical practice

Beyond technical and biological factors, a fundamental methodological limitation pervades the reviewed literature: the overwhelming majority of studies are retrospective, single-institutional, and lack prospective validation in real-world clinical settings. This limitation applies across HCC, BTC, and PDAC applications and represents a critical barrier to clinical translation.

Despite the rapid advancement of artificial intelligence (AI) in digital pathology, its integration into routine clinical practice remains limited (1, 40). Importantly, this persistent gap suggests that the challenge extends beyond technical performance and reflects a fundamental misalignment between algorithm development and real-world clinical needs.

From a technical perspective, data heterogeneity and lack of standardization remain major barriers to model generalizability. Variability in staining protocols, slide preparation, and scanner platforms introduces substantial domain shift, often leading to performance degradation when models are applied across institutions. This highlights a critical translational bottleneck, where models optimized in controlled environments fail to maintain robustness in heterogeneous real-world settings. Although approaches such as stain normalization and domain adaptation have been proposed, robust cross-center validation remains insufficient, particularly for rare hepatobiliary and pancreatic cancers (40, 103). However, current solutions remain largely technical and have not yet translated into standardized clinical workflows. Similar to AI-assisted ultrasonography, digital pathology models remain affected by variability in data acquisition, annotation quality, equipment, operator-dependent factors and limited external validation (104).

Clinical deployment of AI-driven digital pathology is constrained by several practical barriers. First, domain shift arising from variability in scanner platforms, staining protocols, and image acquisition parameters may substantially impair model generalizability, underscoring the need for cross-platform and multicenter validation. From a regulatory perspective, while AI-assisted diagnostic tools have received FDA approval or CE MDR certification for certain cancer types—such as Paige Prostate, approved for prostate cancer in 2021—no AI tool specifically targeting HCC, BTC, or pancreatic cancer histopathology has received clearance from major regulatory authorities to date. These cancers present particular challenges for regulatory validation owing to their high morphological heterogeneity, complex classification systems, and limited availability of high-quality annotated training data. Furthermore, the integration of AI into existing pathological workflows—including infrastructure investment, reimbursement, liability, and pathologist adoption—remains insufficiently addressed in the current literature.

Beyond technical and biological factors, a fundamental methodological limitation pervades the reviewed literature: the overwhelming majority of studies are retrospective, single-institutional, and lack prospective external validation. Retrospective designs introduce selection biases, as cohorts are often enriched for cases with confirmed diagnoses and complete follow-up, which may not reflect the diagnostic uncertainty encountered in routine practice. Single-institutional datasets limit generalizability due to variability in patient demographics, treatment protocols, and pathological processing. Most critically, prospective validation studies remain notably scarce across the reviewed literature, and none describe integration into routine clinical workflows with outcome measurement. This methodological gap represents a critical translational bottleneck that must be addressed before AI-driven digital pathology can be considered clinically reliable in hepatobiliary and pancreatic cancers.

At the model level, the “black-box” nature of deep learning algorithms continues to limit clinical trust and adoption (43). While many models demonstrate high accuracy in retrospective datasets, their decision-making processes often lack transparency. Importantly, current AI systems primarily capture statistical correlations rather than causal biological relationships, raising concerns about their reliability in complex clinical scenarios (26, 43). Consequently, improving interpretability is essential but insufficient without linking model outputs to clinically meaningful mechanisms.This limitation is primarily methodological, reflecting how models learn from data rather than how disease processes are biologically understood.

A promising recent development that directly addresses the generalizability and data scarcity challenges is the emergence of pathology foundation models based on self-supervised pretraining on large whole-slide image corpora. Representative models include UNI, a vision transformer pretrained on over 100, 000 WSIs spanning 20 major cancer types via self-supervised distillation (73); CONCH, a vision-language foundation model trained through contrastive learning between histopathology images and paired pathology reports (74); and Virchow, a large-scale pathology foundation model pretrained on over 225, 000 WSIs and demonstrating robust performance across diverse histopathological tasks (101). These models have demonstrated that pretraining on large multi-cancer, multi-institution datasets enables robust transfer learning to downstream tasks with limited labeled data, achieving performance comparable to or exceeding task-specific models trained from scratch, even when fine-tuned with relatively limited numbers of labeled samples. This paradigm is particularly relevant to HPC: for BTC and PDAC, where single-institution cohorts rarely exceed a few hundred cases, fine-tuning a foundation model requires substantially fewer labeled examples than training from scratch, potentially overcoming the cohort-size bottleneck repeatedly flagged in this review (73, 74, 101, 102). However, while these models have been extensively validated on benchmark datasets, prospective validation in rare cancer settings such as pCCA and PDAC remains an important priority. Future work should focus on adapting these foundation models to HPC-specific challenges, including context-sensitive analysis of cirrhotic backgrounds in HCC and differentiation of heterogeneous histological patterns in BTC and PDAC.

Beyond methodological limitations, a deeper challenge lies in the biological interpretability of AI-derived features. From a biological standpoint, the ability of AI to infer molecular features or tumor behavior directly from histomorphology remains indirect and incompletely understood. Although correlations between morphological patterns and genomic alterations have been reported, the mechanistic links between image-derived features and underlying tumor biology require further elucidation (26, 50). Importantly, without biological interpretability, the clinical credibility of AI-driven predictions remains inherently constrained.

From a clinical perspective, most existing studies remain retrospective and lack prospective validation in real-world settings, which is essential for clinical translation (1, 40). Furthermore, AI models are rarely integrated into existing diagnostic workflows, limiting their practical utility. Rather than replacing pathologists, AI is more realistically positioned as a decision-support tool, yet this role has not been clearly defined or validated in routine practice. From a translational perspective, the key limitation lies in the absence of clearly defined clinical use cases rather than insufficient model performance.

Comprehensive clinical evaluation of AI errors, including their downstream impact on patient outcomes, is essential for safe implementation (105).

A critical dimension of this translational gap is the regulatory and clinical implementation landscape, which remains largely absent from the HPC-specific AI literature. To date, several AI-based digital pathology tools have achieved regulatory approval in oncology broadly: Paige Prostate received FDA breakthrough device designation (2021) and CE marking for prostate cancer detection (106). These approvals demonstrate that the regulatory pathway for AI-driven pathology tools is feasible—yet, based on a review of FDA/EMA regulatory databases and published literature, no equivalent approved tool exists specifically for hepatocellular carcinoma, biliary tract cancer, or pancreatic cancer as of early 2026. This absence is not incidental. HPC presents unique regulatory challenges: the rarity and heterogeneity of BTC and PDAC limit the large, prospectively collected datasets required for regulatory submission (85); HCC frequently arises in the context of underlying liver disease, requiring context-sensitive models that account for cirrhotic backgrounds (5); and regulatory performance thresholds for HPC remain less clearly established than for more common cancers. Moreover, to our knowledge, few if any of the models reviewed in this manuscript have been reported to have entered clinical trials as a primary diagnostic endpoint, received regulatory designation, or been validated in prospective implementation studies. Bridging this gap will require not only technical advances, but active engagement with regulatory frameworks—including pre-submission meetings with FDA/EMA, development of standardized evaluation benchmarks aligned with regulatory requirements, and prospective studies designed explicitly around regulatory endpoints (107).

To illustrate this landscape, **Table 3** summarizes representative AI models in digital pathology for HPCs, stratified into three categories based on their regulatory and translational maturity: (A) Regulatory Approved, (B) Clinically Applied/High Translational Readiness, and (C) In Development/Early-Stage Research, with comparative analysis of their strengths, limitations, and recommended applications for each.

**Table 3:** Representative AI models in digital pathology for hepatobiliary and pancreatic cancers: regulatory and translational status, comparative characteristics, and recommended applications.

Model/system	Cancer type	Primary application	Key performance	Strengths	Limitations	Recommended use
Section A — Regulatory Approved						
Paige Prostate (Paige.AI) (106)	Prostate (reference)	Tumor detection & diagnosis assistance	AUC ~0.99; sensitivity 97.3%	FDA *De Novo* cleared (2021); reduces false negatives	Prostate only; no equivalent for HCC/BTC/PDAC	Regulatory benchmark for HPC AI pathway
Section B — Clinically Applied/High Translational Readiness (multicentre validated; not yet regulatory approved)						
ABRS-P (Zeng et al., Lancet Oncol 2023) (63)	HCC	Treatment response prediction (atezo + bev)	Multicentre retrospec-tive; significant performance	H&E-based; no molecular testing; cost-effective	Retrospective; one regimen; RCT validation pending	Pre-treatment stratification for 1st-line immunotherapy
DL-assisted diagnosis (Kiani et al., NPJ Digit Med 2020) (9)	HCC vs CCA	Histopathologic differential diagnosis	Prospective workflow validation; improved accuracy	Validated AI-assisted workflow; improves consistency	Single-centre; limited external validation	HCC/CCA differential diagnosis support
Foundation models (UNI/CONCH/Virchow) (101, 102)	Pan-cancer (incl. HPC)	Feature extraction; transfer learning	Strong generali-sation; rare cancer performance	Large-scale pre-training; few-shot applicable; rare subtype-friendly	HPC validation insufficient; high compute; limited interpretability	Downstream tasks; rare subtype classification; multicentre research
Section C — In Development/Early-Stage Research (proof-of-concept or single-centre exploratory studies)						
Cheng N et al. (Gastroenterology 2022) (8)	HCC	Hepatic nodular lesion (HNL) classification	AUC 0.90–0.94	Multi-subtype coverage; reduces workload	Small validation set (n=66); single-centre	HNL screening prior to biopsy
Calderaro et al. (Nat Commun 2023) (10)	HCC/ cHCC-CCA	Molecular phenotyping & reclassification of cHCC-CCA	Correlation with molecular profiles	Addresses rare mixed tumour classification; genomics-linked	Small cohort (n=40); prospective data lacking	Mixed hepatic tumour subtype confirmation
Shi JY et al. (Gut 2021) (47)	HCC	Prognostic prediction	Outperforms conventional staging; China & US validated	Weakly supervised; cross-national generalisability	No multimodal integration; interpretability limited	Post-resection risk Stratification
Ding GY et al. (J Hepatol 2022) (76)	BTC/ iCCA	TLS scoring & prognostic stratification	Significant intratumoral vs peritumoral TLS difference	Top hepatology journal; standardises TLS scoring; microenvironment-informed	Needs large-scale validation; complex pipeline	TLS-based immune scoring in iCCA research
Song JW et al. (Sci Rep) (88)	PDAC	Pancreatic cystic lesion classification	AUC 0.96; outperforms biomarkers & cytology	Quantitative cyst characterisation; surpasses conventional tools	Single-centre; external validation required	Risk stratification of indeterminate cystic lesions

HCC, hepatocellular carcinoma; CCA, cholangiocarcinoma; cHCC-CCA, combined hepatocellular-cholangiocarcinoma; BTC, biliary tract cancer; PDAC, pancreatic ductal adenocarcinoma; iCCA, intrahepatic CCA; TLS, tertiary lymphoid structures; H&E, hematoxylin and eosin; AUC, area under the curve; RCT, randomized controlled trial.Section A: tools with formal regulatory clearance. Section B: multicentre-validated models published in high-impact journals, without regulatory approval or clinical deployment. Section C: proof-of-concept or single-centre studies requiring further validation.

**Figure 2:**
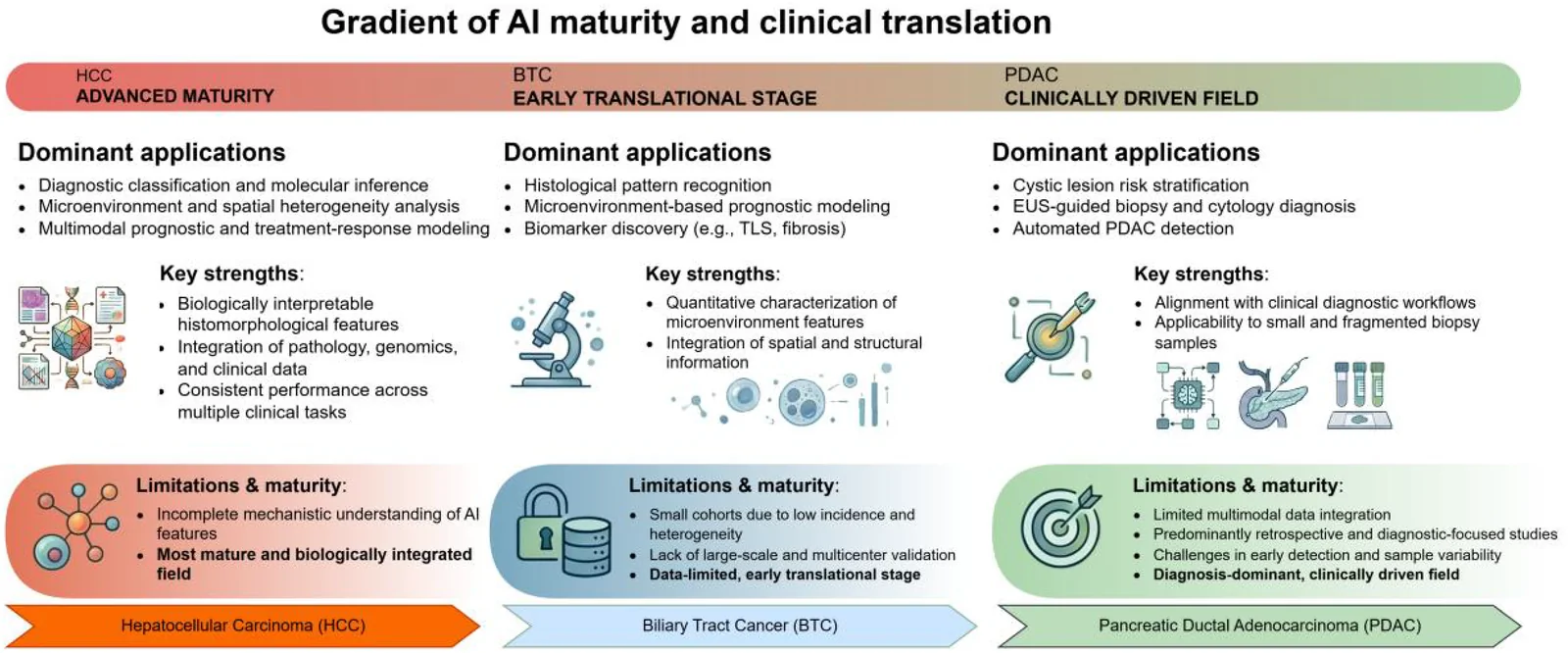
Differential applications and maturity of AI-driven digital pathology across HCC, BTC and PDAC. AI-driven digital pathology shows distinct application patterns across HCC, BTC, and PDAC, structured by dominant applications, key strengths, and current limitations.HCC represents the most advanced and biologically integrated setting, where multimodal models enable prognostic and treatment-response prediction. BTC remains a data-limited and early-stage field, primarily focused on diagnostic assistance and microenvironment-based prognostic features. PDAC is characterized by a clinically driven, diagnosis-oriented paradigm, with emphasis on early detection and biopsy-based analysis.These differences reflect heterogeneous research maturity and highlight the need for disease-specific strategies to facilitate clinical translation. HCC, hepatocellular carcinoma; BTC, biliary tract cancer; PDAC, pancreatic ductal adenocarcinoma; EUS, endoscopic ultrasound; TLS, tertiary lymphoid structures. HCC, hepatocellular carcinoma; BTC, biliary tract cancer; PDAC, pancreatic ductal adenocarcinoma; AI, artificial intelligence; TLS, tertiary lymphoid structures; EUS, endoscopic ultrasound.

Taken together, these limitations explain why AI-driven pathology, despite its promise, has not yet fundamentally transformed clinical decision-making in hepatobiliary and pancreatic cancers. As illustrated in **Figure 2**, AI-driven digital pathology exhibits distinct levels of maturity and translational trajectories across different cancer types, highlighting the gap between technological development and clinical implementation.

### 5.2 From performance to clinical utility: redefining research priorities

The AI applications reviewed in this manuscript span a spectrum of clinical maturity. HCC diagnostic applications, particularly for subtype classification and differential diagnosis, represent the most advanced area, with several models approaching expert-level performance in retrospective settings. In contrast, molecular prediction from histology and spatial heterogeneity analysis remain largely at the proof-of-concept stage. For BTC and PDAC, nearly all AI applications remain at the early research or exploratory stage, with no disease-specific tool having received regulatory clearance to date.

To enable the clinical translation of AI-driven digital pathology, a shift from proof-of-concept studies to clinically oriented research is urgently required. Importantly, this transition reflects a broader need to move from performance-driven development toward clinically meaningful problem definition and solution design. This transition requires not incremental technical improvement, but a redefinition of research priorities toward clinical utility.

First, prospective, multicenter validation studies are essential to establish the robustness and generalizability of AI models (1, 40). Such studies should incorporate diverse patient populations, standardized protocols, and clinically relevant endpoints to ensure real-world applicability. Importantly, validation should move beyond performance metrics toward demonstrating impact on clinical decision-making. In this context, validation should be designed not only to confirm accuracy, but to evaluate real-world clinical benefit and implementation feasibility.

Second, the integration of multimodal data—including histopathology, radiology, genomics, and clinical variables—represents a critical direction for improving model performance and interpretability (49, 50). Multimodal approaches are more likely to capture the complex biological heterogeneity of tumors and align with clinical decision-making processes. Notably, the transition from single-modality models to multimodal frameworks may represent a key step toward clinically meaningful AI applications. From a translational perspective, such integration helps bridge the gap between image-derived features and underlying disease mechanisms. However, such integration should be hypothesis-driven rather than purely data-driven to ensure biological relevance.

Furthermore, pathology foundation models enable data-efficient fine-tuning by leveraging general-purpose representations pretrained on large multi-cancer corpora, offering a practical path to adapt powerful models to small HPC cohorts without sacrificing generalizability (73, 74, 101).

Third, enhancing model interpretability is crucial for building clinical trust. Techniques such as attention mapping, feature attribution, and biologically informed model design may help bridge the gap between computational outputs and pathological reasoning. Developing explainable AI systems that align with established pathological knowledge will be essential for clinical adoption (43). However, interpretability should not be viewed as a purely technical objective, but as a means to support clinical reasoning and decision-making. Nevertheless, interpretability should be aligned with clinical reasoning rather than solely improving visualization of model features.

Current interpretability approaches can be broadly classified into three categories. Attention-based methods, such as Grad-CAM and Class Activation Mapping, visualize spatial regions that contribute most to model predictions, enabling pathologists to verify whether the model focuses on clinically relevant structures. Feature attribution techniques, including SHAP (SHapley Additive exPlanations) and saliency mapping, quantify the contribution of individual features or image patches to final predictions. Concept-based approaches attempt to align latent model features with interpretable pathological concepts, such as nuclear pleomorphism or stromal architecture, facilitating clinical validation of AI-derived patterns.

Despite these advances, significant limitations remain. Attention maps often reflect correlations rather than causal relationships, and may highlight spurious image artifacts rather than genuine pathological features. Feature attribution methods can be unstable across minor model perturbations, raising concerns about their reliability in clinical settings. Most critically, many high-performing AI features remain biologically opaque—they lack clear correspondence to established histopathological markers or molecular mechanisms. Bridging this interpretability gap will require closer collaboration between AI researchers, pathologists, and molecular biologists to develop biologically grounded and clinically actionable explanatory frameworks.

Finally, greater emphasis should be placed on the integration of AI into real-world clinical workflows, including its role in biopsy assessment, intraoperative decision-making, and treatment planning. Defining clear clinical use cases—such as triage, risk stratification, or treatment response prediction—will be critical for successful implementation. This step represents the final and most critical stage of translation, where AI shifts from an analytical model to a clinically embedded decision-support system. Ultimately, the success of AI will depend on its ability to function as an embedded clinical tool rather than an isolated analytical system.

A key frontier is the integration of digital pathology with radiology, genomics, and clinical data to enable multimodal prognostic models. While this review focuses on pathology-centric AI, emerging evidence across HCC, BTC, and PDAC suggests that combining histological features with imaging biomarkers and molecular profiles significantly improves predictive performance. Standardized multimodal frameworks applicable to all three cancer types should be prioritized in future research.

### 5.3 Future perspectives: from tools to decision-making systems

Given the growing global burden of hepatobiliary and pancreatic cancers (85), AI-driven digital pathology may evolve from a research-focused tool to a clinically integrated component of precision oncology. Importantly, this transition reflects a broader paradigm shift from technology-driven innovation toward clinically driven implementation. Advances in whole-slide imaging, deep learning architectures, and computational power will continue to improve diagnostic accuracy and predictive performance. In the coming years, the focus is likely to shift from model development toward real-world deployment and clinical validation.

Importantly, the role of AI could potentially shift from static diagnostic assistance toward dynamic clinical decision support, including treatment selection and longitudinal disease monitoring. This shift signifies a move from isolated analytical outputs to continuous, context-aware clinical support systems. The integration of pathology with emerging technologies such as spatial transcriptomics and radiomics will further enhance the ability to characterize tumor heterogeneity and therapeutic vulnerabilities (49, 50). This convergence of technologies may enable a more comprehensive and mechanistically informed understanding of tumor biology. Future digital pathology systems may combine vision models for WSI analysis with language models for pathology report extraction, data structuring and longitudinal clinical integration (105).

Prospective clinical trials and real-world implementation studies has the potential tovalidate AI systems and defining their clinical value. Beyond validation, such studies will be essential for establishing standardized evaluation frameworks and demonstrating tangible clinical benefit. In parallel, regulatory frameworks and standardized evaluation criteria will be necessary to ensure safe and equitable deployment. Without such frameworks, the translation of AI into routine practice will remain limited despite technical advances.

Ultimately, the success of AI-driven digital pathology will depend not only on technological advancement, but on its ability to deliver measurable clinical benefit and to be seamlessly integrated into real-world clinical workflows. In this context, the future role of AI should be viewed as collaborative rather than substitutive, reinforcing clinician-centered decision processes. The successful integration of AI into hepatobiliary and pancreatic cancer management will depend on sustained multidisciplinary collaboration and a clear focus on clinical utility. Taken together, the future of AI-driven pathology will be defined not by accuracy alone, but by its ability to reshape clinical decision-making in a meaningful and measurable way.

## 6 Conclusion

AI-driven digital pathology is undergoing a paradigm shift from improving diagnostic accuracy to enabling clinically actionable decision support in hepatobiliary and pancreatic cancers. Importantly, this transition reflects a broader movement toward clinically integrated and decision-oriented systems. Despite promising advances, challenges such as data standardization, interpretability, and clinical validation, and regulatory engagement—including alignment with FDA/EMA evaluation criteria—remain significant barriers to clinical implementation. However, these challenges underscore the need for closer alignment between AI development and real-world clinical demands. Future efforts should prioritize prospective validation, multimodal integration, and clinically meaningful endpoints to enhance real-world applicability. Ultimately, the impact of AI-driven digital pathology will depend on its ability to deliver measurable clinical benefit and to be seamlessly integrated into routine practice.
